# The Future of Shift Work: Circadian Biology Meets Personalised Medicine and Behavioural Science

**DOI:** 10.3389/fnut.2020.00116

**Published:** 2020-08-07

**Authors:** Gregory D. M. Potter, Thomas R. Wood

**Affiliations:** ^1^Independent Researcher, London, United Kingdom; ^2^Division of Neonatology, Department of Pediatrics, University of Washington, Seattle, WA, United States; ^3^Division of Human Health, Performance and Resilience, Institute for Human and Machine Cognition, Pensacola, FL, United States

**Keywords:** chronomedicine, chrononutrition, chronotherapy, circadian disruption, light exposure, physical activity, shift work, time-restricted eating

## Abstract

Shift work is commonplace in modern societies, and shift workers are predisposed to the development of numerous chronic diseases. Disruptions to the circadian systems of shift workers are considered important contributors to the biological dysfunction these people frequently experience. Because of this, understanding how to alter shift work and zeitgeber (time cue) schedules to enhance circadian system function is likely to be key to improving the health of shift workers. While light exposure is the most important zeitgeber for the central clock in the circadian system, diet and exercise are plausible zeitgebers for circadian clocks in many tissues. We know little about how different zeitgebers interact and how to tailor zeitgeber schedules to the needs of individuals; however, in this review we share some guidelines to help shift workers adapt to their work schedules based on our current understanding of circadian biology. We focus in particular on the importance of diet timing and composition. Going forward, developments in phenotyping and “envirotyping” methods may be important to understanding how to optimise shift work. Non-invasive, multimodal, comprehensive phenotyping using multiple sources of time-stamped data may yield insights that are critical to the care of shift workers. Finally, the impact of these advances will be reduced without modifications to work environments to make it easier for shift workers to engage in behaviours conducive to their health. Integrating findings from behavioural science and ergonomics may help shift workers make healthier choices, thereby amplifying the beneficial effects of improved lifestyle prescriptions for these people.

## Introduction

Simplistically, our species once lived by two “clocks.” One of these clocks is the environmental clock, which generates roughly 24-h changes in the light/dark (LD) cycle. The other clock is the endogenous biological clock, which among other rhythms generates roughly 24-h (circadian) rhythms in biological outputs such as the sleep/wake cycle. Prior to the introduction of artificial light at night, these two clocks were probably tightly synchronised (1, 2). Following industrialisation, however, people can more easily work outside of conventional daytime hours, and 15–20% of the working population now work shifts (3). The burden of shift work is striking: Shift workers are not only at increased risk of accidents (4), they are also disposed to developing numerous diseases, including certain cancers, coronary heart disease, stroke, and type-two diabetes (5). Few studies have explored whether shift work makes individuals prone to neurodegenerative diseases (6, 7), but shift work frequently disrupts biological rhythms and sleep, and such disturbances propagate a slew of pathobiological changes that contribute to neurodegeneration (8). While at the time of writing little is known about the effects of the 2019 novel coronavirus disease (COVID-19) pandemic on the lives of many shift workers, we would be remiss not to mention that many healthcare professionals at the frontlines of the outbreak are currently working long shifts in conditions that dispose them to developing COVID-19 (9). Many of the chronic conditions associated with shift work are also associated with greater risk of poor outcomes in those with COVID-19 as well as other coronavirus and influenza infections (10–17). As shift workers often work jobs considered essential during the COVID-19 pandemic, improving the health of shift workers should become a key part of current and future pandemic preparedness. Importantly, however, at present there is no strong evidence that people fully adapt to shift work (18). And considering that the unconventional schedules of shift workers also interrupt the lives of cohabiting non-shift workers, the burden of shift work is greater still. The purpose of this manuscript is therefore to summarise some ways by which we might be able to reduce this burden.

## Optimising Shift Work Schedules

Optimising shift work schedules is fundamental to the health and productivity of shift workers. In general, it appears that most shift workers tolerate rapid, forward (clockwise) rotation schedules best (5). To support worker wellbeing, these shifts should each last no longer than 10 h, have at least 11 h of recovery between them, and amount to no more than 60 h of work per week (5). To hone shift work schedules for individual workers, workers may benefit from having some control of their schedules. This autonomy helps account for differences between people in non-work responsibilities, tolerance to shift work, and commuting to and from work.

Chronotype is another tailoring variable that is particularly germane to optimising shift work schedules. Chronotype is defined as interindividual differences in the phenotypic expression of behavioural outputs regulated by the circadian system (19), the most conspicuous of which is the timing of the sleep/wake cycle, and in industrialised societies there exist large differences between individuals in their chronotypes (20). Chronotype appears to modify the association between shift work schedules and risk of health problems (21), such that the health of early chronotypes may be especially negatively affected by working night shifts (22), whereas late chronotypes find working morning shifts particularly problematic (23). While it is not clear precisely why this interaction exists, shift workers who have closer alignment between their chronotypes and their work schedules appear to have more robust melatonin rhythms than their fellow shift workers, suggesting that they have better circadian system function (24). Shift workers who have chronotypes that are better matched to their work schedules may also sleep better (23), and it is increasingly clear that circadian system and sleep health are essential to perhaps all facets of human health (25, 26).

## Optimising Zeitgeber Schedules

The period of each individual's circadian system is one determinant of his or her chronotype. In the absence of time cues (zeitgebers), the free-running period of the human circadian system is slightly longer than 24 h, on average (27). The circadian system therefore needs to be synchronised (entrained) each day with the 24-h day, and shift work complicates this process.

### Exposure to Light

Retinal light exposure is generally regarded as the most important stimulus in entraining the human circadian system (28), and changes in patterns of light exposure can rapidly and substantially shift circadian system timing (phase). This is especially true of short-wavelength light, which most potently suppresses melatonin synthesis (29). Exposure to such light in the biological morning tends to advance circadian phase, whereas exposure to such light in the late biological evening tends to delay circadian phase. The implication of this is that it is possible to bolster how well-shift workers adapt to work schedules through timely use of means to increase exposure to high-intensity, short-wavelength light at specific times of day (e.g., by using light-therapy lamps) and means to reduce exposure to such light at specific times of day (e.g., “blue-blocking” glasses and blue-light filtering apps on electronic devices). While not all studies that have used interventions to modify exposure to light in shift workers have proven beneficial, this inconsistency likely reflects marked heterogeneity in the methods used by researchers (30), as well as large variation between people in how they respond to light (31).

### Melatonin

During darkness, retinal photoreceptors no longer register exposure to light, relaying this to the central clock in the circadian system (the suprachiasmatic nucleus), which in turn signals the pineal gland to synthesise melatonin. Melatonin therefore acts as an endogenous marker of darkness, agonising its receptors in cells in numerous tissues to signal them to fulfil time-of-day-specific functions. Simplistically, when the concentration of melatonin in the blood surpasses a certain threshold in humans who are melatonin-proficient, it is the biological night-time. Conversely, when the concentration of melatonin is below this threshold, it is the biological daytime.

Melatonin supplementation can shift the phase of the circadian system (32). Melatonin ingestion in the late biological afternoon tends to advance circadian phase, while ingestion in the early biological morning tends to delay it. Melatonin is therefore a chronobiotic – an agent that can modify circadian phase. When timed appropriately, light exposure and melatonin ingestion additively shift circadian phase (33).

### Exercise

A growing body of evidence also shows that exercise can shift circadian phase. Early research demonstrated that 15 min of cycling exercise each hour of night shifts helped workers adjust their circadian systems to a 9-h delay in bedtime (34). More recent work has begun to clarify the precise nature of the relationship between exercise and circadian phase, showing that treadmill exercise done in the early biological morning or early biological afternoon advances circadian phase, whereas the same exercise done in the biological evening delays it (35). This relationship is therefore similar to how timing of exposure to light affects circadian phase, and timely exposure to both light and exercise can also additively shift circadian phase (36).

### Nutrition

The influence of timing of food availability on patterns of activity in rats was documented as early as a century ago (37), and numerous studies of such “food anticipatory activity” have since implicated nutrition as an influence on circadian system timing. Whereas the LD cycle is the primary zeitgeber for the suprachiasmatic nucleus, some scientists have hypothesised that the eating/fasting cycle may be the primary time cue for some peripheral clocks in the circadian system. We now know that changing the timing of food consumption rapidly alters the timing of gene transcription in peripheral clocks in mice, for example (38). Recent work has shown that this may be true of humans too, for changing meal timing independently shifts the expression of some genes in peripheral tissues as well as the timing of the blood glucose rhythm, without changing the phase of the melatonin rhythm (39). We acknowledge, however, that lack of control of variables such as LD cycles in most studies of the effects of nutrition on the human circadian system mean that this is arguably the only study of people that fulfils at least one of the criteria for diet to be classified as a zeitgeber (40). It could be that entrainment to LD cycles largely nullifies any zeitgeber effects of nutrition (41).

Summarising the above, it is plausible that carefully timed exposure to light, melatonin ingestion, and exercise may result in additive shifts in the phase of the suprachiasmatic nucleus. As eating/fasting cycles appear to affect the phases of some peripheral circadian clocks, we anticipate that coordinated changes in all of these variables could be used to expedite adaptation to new shift work schedules. If one could estimate shift workers' circadian phases in real time and model how subsequent changes in zeitgeber schedules would influence their circadian systems, one could develop tools that use this information to expedite adaptation to shift schedule changes by providing personalised guidance and perhaps even individual-level changes in exposure to light. This may be a particularly fruitful topic for further study.

## Chrononutrition: The Importance of Diet Timing

While it is plausible that one could change nutrient timing to accelerate adaptation to new shift work schedules, in many instances shift workers do not seek to fully adjust to their new shifts. This raises the question of whether workers undergoing transient changes in work schedules should adjust their diets accordingly. However, it is also crucial to consider contextual factors that influence when shift workers eat and drink. Work schedules, time constraints, timing of breaks within shifts, family commitments, and prioritising behaviours such as sleep over meals all influence diet timing in shift workers, leading to erratic diet timing patterns in these people (42). Diet timing irregularities are also affected by cultural factors (e.g., Ramadan) and the nature of some jobs (e.g., many on-call workers have especially unpredictable work schedules). Temporarily putting these complexities to one side, controlled experiments have begun to explore the effects of diet timing during pre-clinical and clinical simulations of shift work. Table 1 summarises our dietary and supplementation suggestions for shift workers based on our interpretation of the current literature.

**Table 1:** Dietary and supplementation suggestions for shift workers.

The caloric period	Workers should restrict consumption of all items containing > 5 calories to a 6- to 12-h period each day, when possible. They should keep the timing of this period as regular as is feasible from day to day. Workers should self-select the timing of this period, and the ideal time for this period may be relatively early in each worker's biological daytime. We therefore recommend that workers select a caloric period that finishes at least 3 h before their most common bedtime.
Distribution of macronutrient intakes within the caloric period	Workers who have poor cardiometabolic health should aim to consume at least half of daily energy intake in the first half of the caloric period (e.g., by increasing the size of breakfast and reducing the size of dinner). This is less relevant to people who exercise in the second half of their caloric period. Workers should also aim to evenly divide their protein intakes between dietary events. As a starting point, we recommend that workers aim to consume ~ 0.4 g protein per kg bodyweight at each of 3 to 4 evenly-spaced dietary events each day (43).
Sequence of macronutrient intakes within dietary events	Workers who have poor glycaemic control should consume carbohydrate-rich foods last at dietary events, when practical (e.g., consuming fibre- and protein-rich salads before meals or eating meat and vegetable foods before carbohydrate-rich foods).
Snacking outside the caloric period	When workers feel it would be beneficial to snack outside of the caloric period (e.g., to abate hunger and/or support alertness), they may benefit from consuming relatively small (i.e., ~10–20% daily caloric intake), minimally processed, micronutrient-dense, satiating, easy to digest, convenient snacks. We hypothesise that relatively high-protein, low carbohydrate snacks are ideal at these times (e.g., snack items may include boiled eggs, dairy products, minimally-processed fish jerky or meat jerky, high-protein drinks, nuts, whole vegetables, and/or low-sugar whole fruits such as berries).
Caffeine	If their goal is to support cognitive function during shifts, workers may benefit from individual doses of 1–4 mg caffeine per kg bodyweight, favouring the upper end of this range if short on sleep (44). Repeated doses of caffeine every 2 h or so may maximally support cognitive function during extended wakefulness (45). As consuming caffeine as gum leads to faster absorption than consuming caffeine as capsules (46), caffeinated gum may be particularly helpful if the goal is to affect cognition as quickly as possible. Since mistimed caffeine intake impairs sleep, workers should also stop consuming caffeine at least 7 h before the main sleep period, if possible (47). Individuals differ remarkably in their responses to caffeine ingestion, so they should moderate their intakes according to their individual responses. As a starting point, we recommend consuming no more than 6 mg caffeine per kg bodyweight per 24 h.
Creatine	Creatine monohydrate consumption may help shift workers cope with sleep loss. During periods of insufficient sleep, we tentatively recommend that shift workers consume 0.1 g creatine monohydrate per kg bodyweight per day. Because of its potential alertness-boosting properties, we speculate that the ideal time to consume creatine is with the first meal of each day.
Melatonin	Well-timed melatonin use may help some shift workers adapt to new work schedules and sleep better during these transitions. We tentatively recommend that workers consume a dose of 0.3–5 mg melatonin at these times, beginning with a dose at the low end of this range and adjusting the dose according to responses. Because of its potent chronobiotic properties, the optimal timing of melatonin ingestion depends on variables such as the individual's circadian phenotype and work schedule. We therefore do not offer guidance related to melatonin ingestion timing.

### Diet Timing in Shift Workers

Beginning with preclinical research, studies of mice have shown that restricting food access to the dark period (the active phase for these nocturnal animals) may protect against the obesogenic effects of repeated 6-h advances in the LD cycle (48). In addition, restricting food access to the active phase may also accelerate adaptation of circadian rhythms in core body temperature and locomotor activity to repeated 12-h changes in LD cycles (49). These findings imply that people would better cope with rotating shift work if they fixed their eating to the daytime, which is somewhat counterintuitive given that fixing eating time during shifting LD cycles might be expected to uncouple circadian rhythms between the suprachiasmatic nucleus and peripheral clocks. It is, however, intuitive that restricting food access to the active phase may be preferable to restricting it to the rest phase, and findings from initial research on humans support this contention.

Among healthy young men undergoing simulated night shift work for 4 days, those who confined their consumption of calorie-containing foods and drinks (i.e., the caloric period) to between breakfast at 07:00 and dinner at 19:00 had superior post-breakfast glucose tolerance after the intervention compared to men who had dinner at 19:00, a meal at 01:30, and breakfast at 07:00 (50). The group that restricted food intake to the daytime also had superior overnight cognitive function (51). This is especially salient given that many shift workers redistribute their energy intakes into the night when working shifts (52). Additional studies using larger sample sizes and investigating the effects of diet composition on a range of round-the-clock postprandial responses will be instructive.

### Time-Restricted Eating: Findings From Non-shift Workers

Studies of adults undergoing time-restricted eating (TRE) also indicate that optimising nutrient timing is likely to be important to cardiometabolic health, although the participants in these studies have generally not been shift workers. We arbitrarily define TRE as consumption of all calorie-containing items within a period of 12 h or less each day. Conversely, we define intermittent fasting as periodic abstinence from consumption of *any calories for at least 24 h*.

Skipping breakfast is one way to implement TRE, and doing so leads to *late* TRE. While breakfast-skipping is a controversial topic, epidemiologic studies have tended to associate breakfast consumption with lower risk of developing cardiometabolic diseases such as heart disease and type-two diabetes (53, 54). However, controlled studies have not shown large effects of skipping breakfast on cardiometabolic health (55). For example, lean adults who skipped breakfast for 6 weeks inadvertently decreased their daily energy intakes, but this change was compensated by reductions in physical activity energy expenditure, resulting in no changes in energy balance or body composition (56). Skipping breakfast did not affect most measures of cardiometabolic health - the only noteworthy difference between groups was that afternoon glycaemic variability was higher in adults who skipped breakfast. A subsequent study implemented the same intervention but only included obese adults (57). In this study, participants in the breakfast-skipping group expended less energy in the morning, but they did not burn fewer calories over the entire day. Daily energy intake was similar in breakfast-skippers and breakfast eaters, and both groups gained weight during the study. People who skipped breakfast did have higher insulinaemic responses to an oral glucose tolerance test, however. These two rigorous studies show that skipping breakfast minimally affects energy balance but may negatively affect glycaemic regulation and some of its determinants. As sleep timing did not differ between groups, breakfast skipping led to a form of late TRE, so these studies imply that late TRE may not be optimal for some aspects of cardiometabolic health.

Skipping breakfast imposes a relatively late caloric period, and an alternative is to shorten the caloric period by way of skipping dinner or having an early dinner. Several recent carefully controlled experiments have shown that such *early* TRE may exert numerous positive effects on health. The first of these experiments reported that compared with a ~ 12-h daily caloric period for 5 weeks, 5 weeks of early TRE (~ 6-h daily caloric period, finished by 15:00) improved insulin sensitivity, blood pressure, appetite regulation, and a marker of oxidative stress in men who have prediabetes (58). The same group of scientists recently reported that in overweight adults, just 4 days of early TRE reduced mean 24-h blood glucose levels and improved metabolic flexibility, among other benefits (59, 60).

These experiments did not compare early TRE to later TRE while keeping the caloric period fixed, however, and to our knowledge, only one study has done this to date (61). The study in question showed that 7 days of both early (08:00 to 17:00) and late (12:00 to 21:00) TRE improved oral glucose tolerance in men at high risk of developing type-two diabetes, although only early TRE lowered fasting glucose, suggest a small advantage of early TRE (61). While this hypothesis needs careful testing, we believe that early TRE may also enhance diet *composition* by reducing intakes of foods and drinks commonly consumed in the evening, such as processed snacks and alcohol.

Together, these studies support the superiority of relatively *early* TRE in adults who have poor cardiometabolic health. However, non-self-selected TRE schedules may interfere with some social activities and be difficult to adhere to in the context of work schedules and family commitments (62, 63). Letting people self-select their TRE periods helps mitigate these undesirable consequences. Indeed, 12 weeks of self-selected TRE minimised these issues in adults with metabolic syndrome, also reducing daily energy intake and potently improving numerous aspects of cardiometabolic health including bodyweight, waist circumference, and blood pressure (64). Moreover, TRE led to more regular diet timing, which may independently be beneficial for cardiometabolic health (65). Interestingly, TRE also improved sleep timing regularity and increased how often participants self-reported restorative sleep. However, this study was an unblinded, single-arm study with only 19 participants included in the data analysis (64).

Based on existing studies, TRE appears to be a safe strategy that is likely to reduce energy intake, which would be especially beneficial for people who have unavoidably sedentary lifestyles. We hypothesise that fixing the timing of each worker's caloric period within regular hours each day supports metabolic health, and it is plausible that this may be especially important in workers who are subject to unpredictable changes in zeitgebers such as LD cycles (e.g., emergency service workers). We further speculate that each worker's biological daytime is the optimal time at which to fix the individual's caloric period, but self-selection of TRE schedules will help people adhere to TRE and avoid undesirable effects on social and family life. This said, scheduling TRE as early as is practical may maximise the beneficial cardiometabolic effects of TRE.

### Distribution of Energy and Macronutrient Intakes Within the Caloric Period: Findings From Non-shift Workers

While a detailed discussion of this subject is beyond the scope of this review, several recent controlled studies have shown that when daily energy intake is fixed, the distributions of energy and macronutrient intakes *within the caloric period* strongly influence cardiometabolic health. For example, one study divided overweight and obese women into two groups that consumed isocaloric weight loss diets for 12 weeks (66). One group consumed half of their daily energy intakes at breakfast, the other group consumed half at dinner. The group that consumed half at breakfast lost more than twice as much bodyweight, more than twice as many centimetres off their waists, and had greater improvements in oral glucose tolerance. Subsequent work by the same scientists demonstrated that when energy intake is controlled, concentrating energy and carbohydrate intakes early in the day leads to enhanced appetite regulation, weight loss, and dramatic improvements in glycaemic control in adults with type-2 diabetes (67). This builds on research demonstrating that having carbohydrate-rich meals early in the day reduces 24-h glycaemia in adults with impaired fasting glucose and/or impaired glucose tolerance (68).

While these studies highlight the advantages of concentrating energy and carbohydrate intakes relatively early in the caloric period, we note that that intelligent inclusion of physical activity leads to acute improvements in postprandial responses to dietary events such that relatively high energy and carbohydrate intakes late in the biological day may not be so problematic if they bookend exercise (69). And staying on the subject of exercise, there is tentative evidence that distribution of daily protein intake affects skeletal muscle protein synthetic responses to resistance training (70). As muscle protein synthesis is the main determinant of muscle protein balance, it is reasonable to assume that evenly dividing and spacing protein intakes between 3 and 4 daily dietary events may help maximise fat-free mass, a key determinant of cardiometabolic health (43).

### Sequence of Macronutrient Intakes Within Dietary Events: Findings From Non-shift Workers

We would be negligent to not mention that the sequence of macronutrient intakes *within* dietary events may also meaningfully affect postprandial responses. Several studies by one research group have shown that consuming carbohydrate last at a given dietary event (e.g., a full meal) dramatically reduces postprandial glycaemia and insulinaemia in adults who have prediabetes or type-two diabetes (71–73). Shift workers who have poor glycaemic control may hence benefit from consuming carbohydrate-rich foods last at dietary events, when practical.

### Snacking in Shift Workers

Most shift workers snack during night shifts. The problem is that night shifts often occur during the workers' biological night-times, and digestive and metabolic responses to dietary events are impaired during the biological night (74). As highlighted earlier, eating and/or drinking during the biological night-time may disrupt peripheral clocks. If workers snack during night shifts, it is therefore important to minimise energy intake and select dietary choices that lead to favourable postprandial responses. These snacks should also be convenient, minimally processed, micronutrient-dense, satiating, easy to digest, and minimally perishable, when applicable.

Preliminary research has shown that when 24-h energy and macronutrient intakes are controlled during simulated night shifts, a small snack (containing 10% of daily energy intake) may support cognitive function and performance in simulated driving compared with no snacking or a larger meal containing 30% of daily energy intake (75). In this instance, the small snack also reduced hunger to a comparable extent to the meal, without leading to significant digestive discomfort (76). Compared to large night-time snacks, small night-time snacks may also be better for metabolic health. Glycaemic control is relatively easy to measure and predictive of many health outcomes, and some researchers have therefore focused on the effects of nocturnal snacking on glycaemic control. Compared with a small midnight snack (~200 calories), a large midnight snack (~500 calories) impaired postprandial glycaemic responses at a subsequent breakfast at 08:30 during simulated shift work (77). Research such as this is informative, but we again need additional studies of workers in which the effects of dietary changes on metabolic parameters are measured around the clock.

## Chrononutrition: The Importance of Diet Composition

Shift workers are not only apt to consume foods and drinks at suboptimal circadian phases, the quality of shift workers' diets is often worse than that of day workers too. Many shift workers report consuming few fruits and vegetables while also consuming a variety of processed foods at work, such as biscuits, cakes, chocolates, pastries, sandwiches, and fried foods (42). As diet composition affects metabolic health and cognitive function, it is important to help these people make better dietary choices. One way by which diet composition influences health is via effects on the circadian clockwork, and the ketogenic diet (KD) exemplifies this. There has been a resurgence in interest in the KD of late, and while some believe that the restrictive nature of the KD is a barrier to its widespread implementation, certain properties of the KD make it an appealing option for some shift workers who are able to adhere to it.

### The Ketogenic Diet

Studies of mice have shown that the KD has chronobiotic actions on the clocks in multiple peripheral tissues, including the brain, gut, and liver (78–80). Interestingly, Tognini and colleagues found that a KD induced distinct changes in the liver and gut clocks in mice. Compared to a control diet, consumption of a KD produced greater amplitudes of clock gene transcription and their downstream products in the liver, as well as inducing 24-h oscillations in the transcription of many genes in the gut (78). As disruption of the gut clock is associated with increased intestinal inflammation and permeability, as well as endotoxaemia (78, 81), if translatable to humans these results suggest that shift workers who follow a KD may protect themselves against some of the adverse consequences of consuming calories at suboptimal circadian phases.

More generally, both the KD and less severe carbohydrate restriction may reduce some negative effects of shift work on metabolic health. Shift workers are at an increased risk of impaired glucose tolerance and type-two diabetes, and restricting carbohydrate intake is likely to reduce fasting and postprandial glycaemia, both of which are precursory to numerous chronic diseases (e.g., some cardiovascular diseases, certain cancers, and dementia) (82–87). Preliminary evidence has shown that a multicomponent lifestyle intervention centred on the KD may also improve subjective sleep quality in adults who have poor glycaemic control (88), suggesting that sleep enhancement may mediate some of the reported benefits of the KD.

In preclinical studies, ketone bodies themselves have been found to have pleiotropic beneficial physiological effects, including modulation of inflammation, tissue-specific suppression of mTOR signalling, and increased production of brain-derived neurotrophic factor (89–91). If translatable to humans, these systemic effects of ketone bodies imply that long-term consumption of a KD could reduce risk of certain cancers and neurodegenerative diseases such as Alzheimer's in shift workers, particularly those that are already at increased risk (92, 93). Increased production of ketone bodies may also account for some benefits of fasting and TRE. For example, early TRE led to greater morning beta-hydroxybutyrate levels compared to a 12-h caloric period (59). However, there have not yet been any clinical trials of the KD in shift workers, and it will be interesting to explore how the combination of the KD and TRE and/or intermittent fasting interact to affect ketosis, metabolic regulation, and circadian biology in these people.

### Other Dietary Chronobiotics

In addition to effects of dietary patterns on the circadian system, specific dietary compounds have chronobiotic actions. A multitude of dietary compounds affects the circadian system and sleep (94, 95), and it is beyond the scope of this article to discuss them all. We therefore focus on some of those that we anticipate may be practical and beneficial for shift workers. In the future, screens for novel chronobiotics and hypnotics may yield compounds that support the health and performance of these workers (96). Identifying agents that counter decrements in health and cognitive function incited by sleep disruption would also benefit shift workers.

### Caffeine

Largely by antagonising adenosine receptors, consumption of caffeine can improve alertness, attention, reaction time, and mood, as well as physical performance in tests of endurance, strength, and power (44). Studies of caffeine consumption by shift workers have consistently shown beneficial effects on multiple aspects of cognitive function, although whether this results in improved safety is not clear (97). The trade-off is that caffeine consumption tends to prolong sleep latency, reduce slow-wave activity during sleep (which is important to numerous restorative processes), shorten sleep duration, fragment sleep, and worsen subjective sleep quality (98). Consumed late in the day as coffee, caffeine also delays circadian phase (99). Thus it is clear that while judicious caffeine intake can be used to help shift workers perform at work - especially when sleepy - mistimed caffeine intake may strongly degrade sleep, which is noteworthy given that many of the adverse consequences of shift work appear to relate to its detrimental effects on sleep (5). It therefore seems prudent to recommend that shift workers generally stop consuming caffeine several hours before their main sleep period (more specific guidance on caffeine intake is provided in Table 1).

### Creatine Monohydrate

Antagonising adenosine receptors is one way to reduce the accumulation of pressure to sleep (sleep homeostasis), but another is to bolster the phosphorylation of adenosine. Creatine (creatine monohydrate, specifically), a safe and inexpensive dietary supplement that increases brain phosphocreatine stores, countering the accumulation of extracellular adenosine in the brain during extended wakefulness. A study of rats showed that adding creatine to the rats' chow for 4 weeks reduced the duration and slow-wave activity of the rats' sleep (100). We do not currently know the effects of creatine supplementation on sleep in humans, however. Notably, while shorter sleep would generally be expected to impair health and performance, creatine supplementation has repeatedly been shown to *enhance* these variables in humans. Creatine supplementation routinely improves performance in - and adaptations to - many exercise tasks, and creatine has a number of therapeutic actions, including neuroprotective properties (101).

Interestingly, creatine supplementation may also acutely help protect against the deleterious consequences of sleep loss. After sleep loss, creatine supplementation seems to offset deterioration in executive function, mood, reaction time, balance, and other motor skills (102–104). Although we expect creatine supplementation to be a useful strategy to help but this people cope with shift work, we are not aware of any research on this topic. We also note that there is some evidence that concurrent consumption of caffeine may reduce some of the ergogenic effects of creatine on physical performance (105), and additional studies are needed to better identify how the two compounds interact.

### Dietary Amino Acids

Several dietary amino acids may influence circadian rhythms and sleep. For instance, L-tryptophan is a precursor to melatonin that researchers have studied with respect to circadian rhythms and sleep. As an example, there appears to be a temporal relationship between consumption of L-tryptophan in breast milk and infant urinary excretion of 6-sulfatoxymelatonin, the primary metabolite of melatonin (106). Furthermore, infants fed L-tryptophan-enriched night-time formula seem to experience more consolidated sleep/wake patterns (107). Many studies of adults have also shown that ~ 2 g L-tryptophan each day enhances some sleep parameters, although it is not a potent hypnotic (108). To our knowledge, there are no rigorously controlled studies demonstrating that L-tryptophan affects circadian phase, however.

Overall, there has been little research on whether amino acids affect circadian system parameters. In a screen of whether amino acids affect light-induced shifts in the phase of wheel running activity in mice, L-serine increased the magnitude of phase shifts by 86%. This effect seems to translate to humans, as adults who consumed L-serine before bedtime experienced a greater advance in circadian phase in response to bright light exposure (109). Another study reported that 1 week of L-ornithine supplementation delayed the plasma melatonin rhythm by 15 min (110). However, LD cycles and meal timing were not fully controlled in these studies. Interestingly, there is also preliminary evidence that regular L-ornithine supplementation (400 mg per day) may enhance sleep quality during stressful periods (111, 112).

L-glycine may too affect sleep. Consuming 3 g L-glycine an hour before bedtime appears to shorten sleep latency, increase sleep efficiency, and reduce daytime sleepiness in healthy adults, effects that appear to be mediated via the suprachiasmatic nucleus (113, 114). Such supplementation also seems to diminish daytime fatigue and boost vigilance during sleep restriction (115), implying that L-glycine may both enhance sleep and the ability to cope with sleep loss. Given that L-glycine is safe, inexpensive, and may confer other health benefits (116), night shift workers could gain from supplementing with this amino acid. At present, however, there has been little research on effects of this amino acid on sleep.

In summary, it is plausible that supplementing with certain amino acids may help shift workers adapt more quickly to changes in their shifts and/or sleep better, but this is based on few studies that did not control zeitgeber cycles or explore whether the circadian timing of amino acid ingestion interacts with the circadian timing of light exposure. Going forward, it will be important to address these limitations. It will also be interesting to see whether concurrent consumption of different chronobiotic agents additively boosts circadian phase shifts.

## The Future

We have mentioned several ideas for future studies, and we will end by focusing on additional research avenues that may be worth exploring with respect to improving the health of shift workers.

### Using Novel Technologies to Better Personalise Guidance for Shift Workers

Rapid recent advances in the development and uptake of digital technologies such as smartphones, apps, wearables, and artificial intelligence provide scientists with an unprecedented ability to comprehensively assess people's behaviours and health in free-living contexts. The myCircadianClock app is a salient example of such technology. This app has already been used in multiple studies to monitor the circadian phenotypes of study participants, unveiling interesting insights into the effects of interventions such as TRE on human health (64, 117).

As data collected from digital devices are time-stamped, it is easier than ever to temporally map behavioural patterns and their biological sequelae, which could provide novel insights into the causes of changes in the health trajectories of shift workers. One could identify the hours of the day in which it is most frequently a shift worker's biological daytime by longitudinally assessing the timing of the individual's biological clock. As it is not currently practical to assess an individual's melatonin rhythm on a daily basis, the integration of data from surrogate markers of circadian phase such as body temperature and sleep/wake cycles could be used to approximate the timing of the biological daytime. These cycles could be monitored ambiently using data from devices such as smartphones, and the data from the devices could then be used to inform individual shift workers about how to best implement TRE. Where feasible, this process could be refined with the addition of round-the-clock measures of metabolic regulation, such as continuous glucose monitoring.

**Figure 1:**
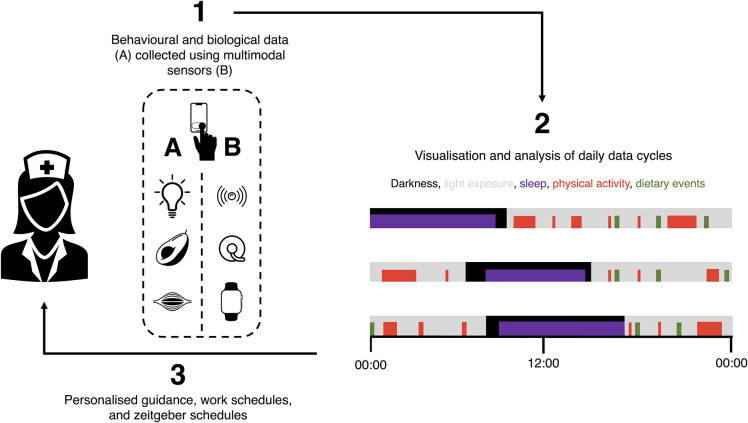
Using digital devices to optimise the health and performance of shift workers by providing personalised guidance, work schedules, and zeitgeber schedules. (1) Digital devices can be used to monitor behaviours and their biological responses, including (i) exposures to variables such as light (e.g., via head-worn wearables), (ii) dietary behaviours (e.g., via food photography and continuous glucose monitoring), and (iii) physical activity (e.g., via smart watches). Digital phenotyping using patterns of smartphone use can be used to enrich this analysis. (2) These data and their interactions can then be analysed in real time and used to (3) personalise guidance, shift schedules, and zeitgeber schedules of individual workers. Guidance can be delivered digitally and in-person, and innovative technologies may eventually allow the automation of adjustments to individual workers' zeitgeber schedules.

At a small scale, the feasibility of this type of approach has already been shown (118). Ultimately, implementing such methods at a large scale and including both shift workers and non-shift-working controls may help develop models that forecast transitions in the health of shift workers, as well as how to alter these trajectories. However, the data collection process will need to be relatively frictionless (for participants, at least) to achieve this. This will be facilitated by close collaboration between scientists and workers in the technology sector. With accurate monitoring in place, digital tools could then be implemented to improve the health and productivity of shift workers by optimising variables such as zeitgeber schedules in real time (Figure 1).

Innovative technologies could also provide novel means of generating insightful data while minimising participant burden. For example, sensors commonly built into smartphones can now be used to monitor blood parameters such as haemoglobin that once required invasive testing (119). Smartphones can also be used to monitor some exposures that are particularly relevant to shift workers, such as patterns of locomotion and exposure to light. One problem, however, is that it would be especially useful to assess exposure to light at the level of the eye. This requires new wearable devices, for smartphones are not suited to this, and many existing wearables that measure light exposure are frequently obstructed by clothing, confounding their data. It is possible to make smart eyewear to estimate retinal light exposure, and such eyewear may be especially useful for another purpose. The utility of all of these monitoring technologies may be enhanced by the addition of the ability to digitally “envirotype” individuals, ambiently tracking information about their environments to better understand the interaction between environment and phenotype (120). Building camera technology into eyewear is one way to accomplish this.

Meanwhile, digital phenotyping – assessing changes in people's phenotypes using data from digital devices – has already been used to identify patients' disease trajectories in neurological disorders such as schizophrenia (121). Such phenotyping can proceed without active user engagement, and it can also be used to assess behaviours such as sleep (122). Ultimately, use of multimodal novel sensors that analyse biofluids including interstitial fluid (e.g., continuous glucose monitoring), saliva, sweat, and tears may prove particularly useful in monitoring variables such as dietary intakes and associated changes in metabolites (123). However, the development of these sensors poses substantial challenges related to biofouling, accuracy, power, usability, calibration, and data security.

These tools are promising approaches to forecasting changes in behaviours and health, and we hope they will help healthcare professionals intervene before individuals succumb to disease. We foresee that using sophisticated computational methods such as deep learning to concurrently analyse individuals' behavioural, health, and environmental data from multimodal sources will eventually enhance personalisation of guidance for individual shift workers (124).

### Applying Behavioural Science to Support Better Health Decisions by Shift Workers

Even if shift workers understand precisely which behaviours they should enact to improve their health, they are prone to a variety of factors that impair decision making, such as circadian system misalignment and sleep loss (125, 126). Furthermore, knowledge alone is rarely sufficient to support lasting health behaviour change (127). It is therefore imperative to support the ability of these people to make smart decisions, and this requires applying principles from behavioural science, particularly at the level of the organisations that employ shift workers.

Significantly, many new technologies are strikingly habit-forming, and this exemplifies the power of applying behavioural science principles to shape behaviour. If scientists and technologists can collaborate to effectively use behavioural science to create engaging, scalable products that deliver tailored health guidance to shift workers, all would benefit. We believe that technologies that deliver adaptive interventions to both help people avoid poor health decisions during states of vulnerability *and* support good health decisions during states of opportunity will be particularly advantageous (128).

The built environment also affects health in numerous ways (129), and given that shift workers are prone to health problems, it is particularly critical to pay attention to optimising the workplaces of these people. As shift workers commonly experience circadian system disruption and do not gain tolerance to such disruption (130), it may be valuable to create workplaces that allow close control over exposure to light, and intelligent use of “smart” lighting systems may benefit these individuals. We also anticipate the development of closed-loop devices that will personalise light exposure at the level of the individual.

The built environment influences physical activity. To support job performance and health, workplaces should have designated exercise spaces to encourage physical activity. Environmental design is relevant to nutrition too. Dietary choices depend strongly on where foods and drinks are sourced from. In work settings such as airplanes, food is provided for shift workers. However, most shift workers are left to source their own food, and when short on time many shift workers buy foods and drinks from vending machines (42). It is encouraging that many workers do select healthier dietary choices when they are available in vending machines, providing an opportunity for organisations to positively affect their employees' health decisions (131). Furthermore, workplace interventions to promote healthier diets, such as offering free fruit and labelling meals, have sometimes been shown to facilitate healthy dietary choices (132, 133). Simple changes in the placement of food in eating areas affect food selection too (134), and these changes can be leveraged to support the health of shift workers. Similarly, if workers are using products such as melatonin supplements and blue-light-blocking glasses to shift the phases of their circadian systems, it makes sense to help them acquire efficacious products.

It is also clear that social life is a strong influence on many shift workers' health behaviours, including their diets. The dietary attitudes and preferences of co-workers affect some workers' dietary choices (135), so group commitment to healthier dietary choices may aid the adoption of more nutritious diets. As stress strongly affects dietary choices in many people and shift workers often report high stress and abnormal dietary behaviours (136), interventions to nurture the resilience of shift workers and to improve workers' self-regulation skills may support their dietary choices. Such interventions include mindfulness-based approaches (137). Shift workers could also benefit from other types of social support, including provision of additional childcare, as well as groups and events designed to minimise conflicts between their work and non-work activities.

Educating shift workers about how to sleep better is likely to be pivotal to their well-being, and shift work workplaces should have spaces for sleepy workers to nap. It is of course important to identify workers who have sleep disorders too, and simple screening tools such as brief questionnaires can be used for this (138). It may too be useful to screen for people who are simply not suited to certain shift schedules, for people differ substantially in how they tolerate shift work. Certain characteristics associate with better shift work tolerance, including robust general health; young age; male sex; not having children; low languidity and neuroticism; high extraversion, flexibility in sleeping habits, and internal locus of control; and a chronotype that is neither very early nor very late (18). Promisingly, personalising shift work schedules by removing night shifts for early chronotypes and excluding morning shifts for late chronotypes has been shown to prolong self-reported sleep, improve subjective sleep quality, and enhance worker well-being (139). To estimate chronotype, a study by Vetter and colleagues used a shift work-specific version of the Munich Chronotype Questionnaire (140), and this approach may be useful to help personalise work schedules for shift workers. Nonetheless, it would be useful to develop additional questionnaires designed specifically to identify appropriate shift schedules, as well as to track how workers respond to these schedules.

Finally, it is worth noting that many workplace wellness programmes that have been tested have not yielded impressive results (141). Assessing the effects of workplace interventions is difficult for numerous reasons, not the least of which are enforcing blinding and randomisation of participants. To date, marked heterogeneity between studies has made it challenging to assess the utility of workplace interventions for shift workers (30). And as is so often the case, the participants included in many of these studies did not comprise a diversity of ages and races, nor did the scientists attempt to define determinants of which workers responded positively to the interventions. None of this means that it is not possible to implement effective programmes, however, and we hope that lacklustre results to date do not stymie continued efforts to improve on workplace interventions by better incorporating principles from behavioural science.

### Using Alternatives to Traditional Study Designs to Better Personalise Guidance for Shift Workers

To assess the efficacy of interventions to improve shift-worker health, it may make sense to use alternatives to many of the hitherto-used study designs. Recently, studies applying “Big Data” approaches have contributed to some advances in efforts to personalise medicine. However, it may be advantageous to concurrently carry out studies that use a “Small Data” paradigm – for example, using *n*-of-1 approaches to more rapidly assess how individual workers are responding to a given intervention and to forecast which of them are at risk of health trajectory transitions towards disease (142).

## Conclusions

A large proportion of the workforce works shifts, and these individuals are integral to sustaining functional societies. However, the study of how to support the long-term health and well-being of these people has been somewhat neglected, and a relatively small proportion of relevant studies has included shift workers as participants. While the type of personalised interventions to support shift workers that we have discussed in this article are bound to produce logistical headaches for employers, the onus should be on supporting the long-term the health and performance of their employees. The acute difficulties arising from implementing customised shift schedule systems and suchlike may be more than made up for by the lasting benefits of these systems on health, safety, and productivity. We note also that as shift work increases the likelihood of adverse pregnancy outcomes and may lead to epigenetic modifications in parents that could plausibly affect the epigenetics and hence health of their children, supporting the health of shift workers could one day have critical effects on the well-being of future generations (143, 144).

Scientists now have an unprecedented ability to identify ways of helping shift workers. We hope that this ability is realised in the near future.

## Author Contributions

GP conceived the idea and drafted the manuscript. TW edited the manuscript and produced the final draft. All authors approved the final version and performed the literature review.

## Conflict of Interest

The authors declare that the research was conducted in the absence of any commercial or financial relationships that could be construed as a potential conflict of interest.
